# Alpha-Enolase: Emerging Tumor-Associated Antigen, Cancer Biomarker, and Oncotherapeutic Target

**DOI:** 10.3389/fgene.2020.614726

**Published:** 2021-01-28

**Authors:** Frankis A. Almaguel, Tino W. Sanchez, Greisha L. Ortiz-Hernandez, Carlos A. Casiano

**Affiliations:** ^1^Center for Health Disparities and Molecular Medicine, Department of Basic Sciences, Loma Linda University School of Medicine, Loma Linda, CA, United States; ^2^Loma Linda University Cancer Center, Loma Linda, CA, United States; ^3^Department of Medicine, Division of Rheumatology, Loma Linda University Health, Loma Linda, CA, United States

**Keywords:** alpha-enolase, autoantibodies, cancer biomarker, therapeutic target, ENO1

## Abstract

Alpha-enolase, also known as enolase-1 (ENO1), is a glycolytic enzyme that “moonlights” as a plasminogen receptor in the cell surface, particularly in tumors, contributing to cancer cell proliferation, migration, invasion, and metastasis. ENO1 also promotes other oncogenic events, including protein-protein interactions that regulate glycolysis, activation of signaling pathways, and resistance to chemotherapy. ENO1 overexpression has been established in a broad range of human cancers and is often associated with poor prognosis. This increased expression is usually accompanied by the generation of anti-ENO1 autoantibodies in some cancer patients, making this protein a tumor associated antigen. These autoantibodies are common in patients with cancer associated retinopathy, where they exert pathogenic effects, and may be triggered by immunodominant peptides within the ENO1 sequence or by posttranslational modifications. ENO1 overexpression in multiple cancer types, localization in the tumor cell surface, and demonstrated targetability make this protein a promising cancer biomarker and therapeutic target. This mini-review summarizes our current knowledge of ENO1 functions in cancer and its growing potential as a cancer biomarker and guide for the development of novel anti-tumor treatments.

## Introduction

Alpha-enolase (ENO1, 47 kD) has recently emerged as a major driver of tumor metabolism and progression and is considered a rising cancer biomarker and therapeutic target (Capello et al., 2011; Hsiao et al., 2013; Principe et al., 2017; Cappello et al., 2018). ENO1 is one of three enolase isoforms encoded by different genes: ENO1, expressed in most human tissues and upregulated in cancer cells; gamma-enolase (ENO2), expressed in neuronal cells and neuroendocrine differentiated tumors; and beta-enolase (ENO3), expressed in muscles (Pancholi, 2001; Isgrò et al., 2015; Ji et al., 2016). These isoforms show high sequence conservation and similar size, and combine to catalyze the dehydration of 2-phosphoglycerate to phosphoenolpyruvate during glycolysis. In cancer cells, this reaction occurs under both aerobic and anaerobic glycolysis, contributing to the Warburg Effect, which increases glucose uptake, proliferation, and tumor growth (Pancholi, 2001; Liberti and Locasale, 2016; Qian et al., 2017).

Alpha-enolase is overexpressed in multiple human cancer types, contributing to increased glycolysis and tumor growth (Altenberg and Greulich, 2004; Chang et al., 2006; He et al., 2007; Tsai et al., 2010; Capello et al., 2011; Song et al., 2014; Fu et al., 2015; Sun et al., 2017, 2019; Zhan et al., 2017; Yin et al., 2018; Zhang et al., 2018, 2020; Cheng et al., 2019; Ji et al., 2019; Qiao et al., 2019; Xu et al., 2019; Chen et al., 2020). ENO1 overexpression is often associated with anti-ENO1 autoantibody responses and may have prognostic and diagnostic value in certain cancers (Table 1; Adamus et al., 1998; Tomaino et al., 2011; Pranay et al., 2013; Hsiao et al., 2015; Griggio et al., 2017; Zhang et al., 2020). ENO1 is also localized on the surface of cancer cells where it enhances plasmin formation (Miles et al., 1991; Redlitz et al., 1995) to promote extracellular matrix degradation, cell migration, invasion, and metastasis (Hsiao et al., 2013; Didiasova et al., 2014; Principe et al., 2015, 2017; Zakrzewicz et al., 2018). These properties make ENO1 a tumor-associated antigen (TAA) and promising cancer biomarker and therapeutic target. Below we summarize ENO1’s functions in cancer, growing potential as a cancer biomarker, and rising opportunities for targeting this enzyme for cancer treatment.

**TABLE 1:** Potential prognostic and diagnostic value of ENO1 expression in tumors and cancer-associated anti-ENO1 autoantibodies.

**Cancer type**	**Molecule**	**Prognostic/diagnostic value**	**References**
Bladder	ENO1	Prognostic	Ji et al., 2019
Breast	ENO1	Prognostic	Tu et al., 2010; Cancemi et al., 2019
Cancer-associated retinopathy	Autoantibodies	Prognostic (progressive blinding)	Adamus et al., 1998
Chronic lymphocytic leukemia	Autoantibodies	Prognostic	Griggio et al., 2017
Colorectal	ENO1	Prognostic	Zhan et al., 2017
Gastric cancer	ENO1	Prognostic	Qian et al., 2017; Qiao et al., 2019; Sun et al., 2019; Xu et al., 2019
Glioma	ENO1	Prognostic	Song et al., 2014
Head and Neck	Both ENO1 and autoantibodies	Prognostic	Tsai et al., 2010; Pranay et al., 2013
Liver	Both ENO1 and autoantibodies	Prognostic/diagnostic	Takashima et al., 2005; Hamaguchi et al., 2008; Zhang et al., 2020
Lung Cancer	Both ENO1 and autoantibodies	Prognostic/Diagnostic	Chang et al., 2006; He et al., 2007; Shih et al., 2010; Hsiao et al., 2015; Dai et al., 2017; Zhang et al., 2018; Zang et al., 2019
Multiple myeloma	ENO1	Prognostic	Ray et al., 2020
Non-Hodgkin’s Lymphoma	ENO1	Prognostic	Zhu et al., 2015
Pancreatic cancer	Both ENO1 and autoantibodies	Prognostic	Tomaino et al., 2011; Sun et al., 2017; Yin et al., 2018; Wang et al., 2019

## Multifunctional Oncoprotein

**FIGURE 1:**
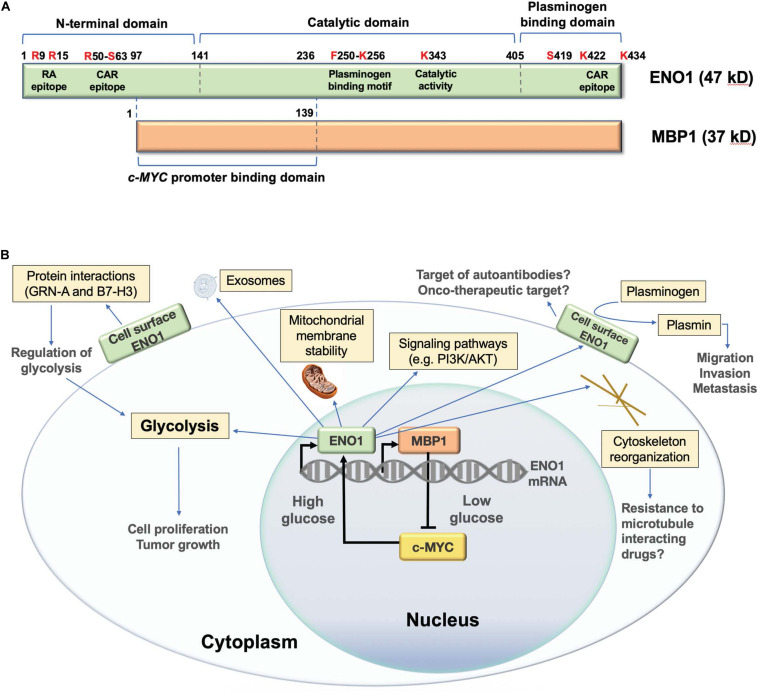
Structure and functions of human ENO1. **(A)** Schematic representation of domain structure of ENO1 and its alternative translation variant c-MYC promoter binding protein 1 (MBP1). Several lysine residues (K256, K422, and K434) have been implicated in the plasminogen binding functions of ENO1, whereas K343 has been implicated in its catalytic activity, required for the conversion of 2-phosphoglycerate to phosphoenolpyruvate during glycolysis. Citrullination of arginines 9 (R9) and 15 (R15) generates an immunodominant peptide (residues 5–22) that is targeted by autoantibodies in patients with rheumatoid arthritis. Methylation of arginine 50 (R50) has been implicated in ENO1 externalization. R50 is also part of an immunodominant epitope recognized by autoantibodies from patients with cancer associated retinopathy (CAR). Another CAR epitope is located within the plasminogen binding domain. Phosphorylated serine 419 (S419) within the plasminogen binding domain is recognized of ENO1 autoantibodies in pancreatic cancer patients. While MBP1 shares the catalytic and plasminogen binding domains of ENO1, it lacks these functions due to its exclusive nuclear localization. MPB1 residues 1–139 (ENO1 96–236) comprise its DNA binding domain, required for binding to the *c-MYC* gene promoter, which results in repression of promoter activity and downregulation of c-MYC protein expression. **(B)** Schematic representation of the cellular functions of ENO1 and MBP1. MBP1 localizes primarily in the cell nucleus where it represses the *c-MYC* gene promoter, whose activity is essential for ENO1 upregulation. ENO1 is primarily localized in the cytoplasm, where it functions in glycolysis, promoting mitochondrial stability and cytoskeleton reorganization, and regulating oncogenic signaling pathways. This protein is also localized on the cell surface, where it acts as a plasminogen receptor and interacting partner of various proteins to regulate glycolysis, as well as cancer cell migration, invasion, and metastasis. ENO1 can also be secreted from cells as a component of exosomal vesicles.

Alpha-enolase mRNA gives rise to an alternative translation product of 37 kD called *c-MYC* promoter binding protein 1 (MBP1) (Figure 1A; Subramanian and Miller, 2000). Although MBP1 does not have glycolytic activity, it regulates the cellular response to altered glucose concentration (Sedoris et al., 2007). ENO1 is upregulated by the c-MYC oncoprotein (Osthus et al., 2000), and is localized in the cytoplasm and the cell surface, playing multiple roles (Figure 1B; Diaz-Ramos et al., 2012; Didiasova et al., 2019). In contrast, MBP1 is a nuclear protein that represses c-MYC transcription under cellular stress and low glucose conditions, leading to decreased cell proliferation (Feo et al., 2000; Subramanian and Miller, 2000; Sedoris et al., 2007; Maranto et al., 2015). The ratio of ENO1/MBP1 expression in cancer cells is regulated by glucose, with c-MYC-driven elevated ENO1 expression under high glucose conditions, and elevated MBP1 expression under low glucose conditions (Sedoris et al., 2007). Cancer cells adapt to hypoxia by overexpressing c-MYC, which stimulates glycolysis and cell proliferation *via* ENO1 upregulation and MBP-1 downregulation (Sedoris et al., 2010). The ENO1/MBP-1 ratio influences cancer aggressiveness, as demonstrated in human breast tumors where overexpression of ENO1 and extracellular matrix metalloproteinases MMP-2 and MMP-9, concomitant with MBP-1 downregulation, correlates with worse prognosis (Cancemi et al., 2019).

Alpha-enolase also plays important roles as a plasminogen receptor, component of exosomal vesicles, cytoskeleton reorganizing protein, stabilizer of mitochondrial membrane, and modulator of oncogenic signaling pathways (Figure 1B; Diaz-Ramos et al., 2012; Didiasova et al., 2019). These functions allow overexpressed ENO1 to promote cancer cell proliferation, survival, clonogenicity, epithelial-mesenchymal transition (EMT), chemoresistance, extracellular matrix degradation, migration, invasion, and metastasis. These functions can be inhibited in cancer cells by ENO1 depletion (Georges et al., 2011; Song et al., 2014; Fu et al., 2015; Zhu et al., 2015; Capello et al., 2016; Principe et al., 2017; Qian et al., 2017; Zhan et al., 2017; Qiao et al., 2018a, 2019; Ji et al., 2019; Sun et al., 2019; Wang et al., 2019; Xu et al., 2019; Santana-Rivera et al., 2020), or targeting with antibodies (Hsiao et al., 2013; Principe et al., 2015), microRNA (miR) (Liu et al., 2018), or long non-coding RNAs (lncRNAs) (Yu et al., 2018). ENO1 also regulates oncogenic signaling pathways, including PI3K/Akt (Fu et al., 2015; Sun et al., 2019; Chen et al., 2020; Zang et al., 2020), v/β-3 integrin (Principe et al., 2017), β-catenin (Ji et al., 2019), transforming growth factor beta (Xu et al., 2019), AMPK/mTOR (Zhan et al., 2017; Dai et al., 2018), and others (Huang et al., 2019).

Acting as a plasminogen receptor, ENO1 “moonlights” on the surface of tumor cells to facilitate plasminogen conversion into plasmin (Miles et al., 1991; Redlitz et al., 1995; Capello et al., 2011; Diaz-Ramos et al., 2012; Ceruti et al., 2013; Hsiao et al., 2013; Didiasova et al., 2014, 2019). During inflammatory conditions, plasmin activation leads to fibrinolysis and facilitates extracellular matrix degradation, a function linked to ENO1’s ability to promote cancer cell migration, invasion, and metastasis (Hsiao et al., 2013; Kumari and Malla, 2015). Bacteria and immune cells take advantage of ENO1’s plasminogen receptor functions to facilitate tissue invasion (Wygrecka et al., 2009; Bergmann et al., 2013).

The plasminogen binding activity of ENO1 has been mapped to the C-terminal peptide _422_KFAGRNFRNPLAK_434_, Miles et al. (1991) and Redlitz et al. (1995), with another putative plasminogen binding site located at _250_FFRSGK_256_ (Figure 1A; Kang et al., 2008). ENO1 surface localization is guided by post-translational modifications (PTMs), particularly methylation of arginine 50 (Zakrzewicz et al., 2018). Other PTMs, including citrullination (Lundberg et al., 2008), acetylation, and phosphorylation (Zhou et al., 2010; Capello et al., 2011; Tomaino et al., 2011; Sanchez et al., 2016), are also likely to influence ENO1 functions, localization, and immunogenicity (Didiasova et al., 2019). ENO1 exteriorization is promoted by lipopolysaccharide (Zakrzewicz et al., 2018), calcium influx (Didiasova et al., 2015), and interaction with caveolin 1, annexin 2, and heat shock protein 70 (Zakrzewicz et al., 2014; Perconti et al., 2017).

Alpha-enolase interacts in the cell surface with B7-H3, an immune co-stimulatory molecule with oncoprotein functions, to promote glycolysis (Zuo et al., 2018). It also interacts with granulin A (GRN-A), a 6 kDa peptide derived from progranulin that inhibits ENO1’s ability to promote cancer cell proliferation, migration, and invasion (Chen et al., 2017). GRN-A synergizes with cisplatin to induce apoptosis in hepatocellular carcinoma cells (Qiao et al., 2018b). Overexpressed ENO1 promotes resistance to cisplatin and other anti-tumor drugs in cancer cells by increasing glycolysis and cell proliferation (Tu et al., 2010; Qian et al., 2017; Qiao et al., 2018a; Wang et al., 2019; Santana-Rivera et al., 2020), interaction with microtubules (Georges et al., 2011), and cell adhesion (Zhu et al., 2015; Principe et al., 2017).

Alpha-enolase has also been implicated in the regulation of T cell effector functions, including the suppressive functions of induced regulatory T cells (De Rosa et al., 2015), T cell activation La Rocca et al. (2017), and the diabetogenic functions of islet-specific CD4+ T cells (Berry et al., 2015). Gemta et al. (2019) recently reported that downregulation of ENO1 activity represses the glycolytic activity of tumor infiltrating CD8+ lymphocytes (CD8+ TILs), leading to their functional exhaustion. This impaired ENO1 function is unrelated to its expression, suggesting the involvement of post-transcriptional regulatory mechanisms such as PTMs influencing ENO1 enzymatic activity or subcellular localization (Gemta et al., 2019).

## Tumor-Associated Antigen

The presence of anti-ENO1 autoantibodies is well documented in autoimmune diseases such as rheumatoid arthritis (RA) and autoimmune retinopathy (Adamus, 2017). In RA, these autoantibodies recognize an immunodominant citrullinated peptide within the ENO1 N-terminus (Lundberg et al., 2008), and are clinical diagnostic biomarkers.

Alpha-enolase autoantibodies are also present in cancer patients, often associated with cancer-associated retinopathy (CAR) (Adamus, 2017). Unlike in RA, ENO1 autoantibodies from CAR patients do not specifically target citrullinated peptides but rather recognize several epitopes, including an immunodominant N-terminal domain peptide, _56_RYMGKGVS_63_, and a C-terminal peptide implicated in plasminogen binding, _421_AKFAGRNF_428_ (Adamus et al., 1998). CAR-linked ENO1 autoantibodies promote retinopathy by inducing retinal cell apoptosis, leading to retinal dysfunction or degeneration (Adamus, 2018; Adamus et al., 2020). *In vitro* treatment of retinal cells with an anti-ENO1 monoclonal antibody significantly impaired glycolysis, reduced ATP production, and induced apoptosis (Magrys et al., 2007). ENO1 autoantibodies from patients with autoimmune retinopathy also target retinal ganglion cells and induce apoptosis in rats (Ren and Adamus, 2004). Further, the survival of retinal cells treated with ENO1 autoantibodies from patients with autoimmune retinopathy and CAR was impaired compared to retinal cells exposed to sera from healthy controls (Adamus et al., 1998). While ENO1 autoantibodies are known to trigger pathological effects through their internalization by retinal cells (Ren and Adamus, 2004), it cannot be ruled out that they also directly target cell surface ENO1, leading to glycolysis impairment and apoptosis. The study of ENO1 autoantibodies in CAR has uncovered a potential unintended consequence - i.e., antibody-induced retinal apoptosis- that requires careful consideration as ENO1-based cancer immunotherapies are developed.

Alpha-enolase autoantibodies are associated with either improved or poor tumor patient outcomes in different cancer types, suggesting a context-dependent clinical significance (Table 1; Shih et al., 2010; Tomaino et al., 2011; Pranay et al., 2013; Hsiao et al., 2015; Griggio et al., 2017). While these autoantibodies may occur in cancer patients independent of CAR, a recent study showed that vision loss and anti-retinal autoantibodies occur in at least 20 different human cancers, with ENO1 being the most frequent target of these antibodies (Adamus et al., 2020). Autoantibodies to other glycolytic enzymes have also been detected in CAR patients (Adamus et al., 2020), suggesting that they are induced by immune presentation of peptides from overexpressed metabolic proteins released from tumor cells (Adamus et al., 2020).

ENO1 autoantibodies have been included in TAA panels for cancer immunodiagnosis. For instance, Zang et al. (2019) examined a panel of four cancer biomarkers (carcinoembryonic antigen, cancer antigen 125, Annexin A1 autoantibodies, and ENO1 autoantibodies) for lung cancer detection that yielded high specificity, sensitivity, and diagnostic accuracy. Dai et al. (2017) also reported that combining ENO1 autoantibodies with carcinoembryonic antigen and cytokeratin 19 fragment in a diagnostic panel increased diagnostic sensitivity for non-small cell lung cancer. Another study detected ENO1 autoantibodies at higher frequencies in patients with early stage lung cancer compared to late stage patients (Zhang et al., 2018).

Post-translational modifications contribute to the generation of ENO1 autoantibodies, as evidenced by the observation that patients with pancreatic ductal adenocarcinoma (PDA) produce antibodies that specifically target epitopes containing phosphorylated serine 419 within the plasminogen binding domain of ENO1 (Figure 1), and correlate with improved outcome in patients receiving chemotherapy, suggesting a protective role (Tomaino et al., 2011). It is not clear if, like in RA, citrullination triggers ENO1 autoantibodies in cancer patients, although citrullinated ENO1 was reported to elicit anti-tumor CD4+ T responses in murine tumor xenografts and in ovarian cancer patients (Cook et al., 2018; Brentville et al., 2020). Our group and others identified citrullinated ENO1 in cancer cells (Jiang et al., 2013; Sanchez et al., 2016), suggesting that this PTM could potentially trigger ENO1 autoantibodies in cancer patients.

We reported that ENO1 autoantibodies occur at higher frequency in prostate cancer (PCa) patients compared to controls, showing racial differences in reactivity (Sanchez et al., 2016). While autoantibodies from European American (EA) PCa patients reacted strongly with human recombinant ENO1 by ELISA but weakly by immunoblotting against endogenous ENO1 from PCa cells, autoantibodies from African American (AA) patients showed the opposite pattern. ENO1 autoantibodies from AA patients also displayed differential reactivity against endogenous ENO1 in a panel of PCa cell lines, reacting strongly with ENO1 in metastatic PCa cell lines by immunoblotting, whereas autoantibodies from EA patients reacted uniformly against this protein across the panel. Intriguingly, ENO1 autoantibodies from AA patients lost immunoreactivity in docetaxel-resistant cells, while autoantibodies from EA patients retained this reactivity. Proteomics analysis revealed differences in PTMs (e.g., acetylation, methylation, phosphorylation, and citrullination) within endogenous ENO1 between chemosensitive and chemoresistant PCa cells, suggesting that the observed racial differences in ENO1 autoantibody reactivity in these cell types might be influenced by PTMs.

In addition to ovarian cancer (Brentville et al., 2020), T cell responses targeting ENO1 have also been reported in patients with PDA. ENO1-specific CD8+ T cell responses were detected in 8 out of 12 PDA patients with circulating anti-ENO1 IgG autoantibodies, whereas patients without these autoantibodies lacked these responses, suggesting an integrated humoral and cellular anti-ENO1 response (Cappello et al., 2009). A later study reported that phosphorylated ENO1 also triggers CD4+ T cell responses in PDA patients (Capello et al., 2015).

## Cancer Biomarker and Therapeutic Target

The need for new cancer-specific targets that can act as beacons to localize tumors with high efficiency is a key feature of a robust biomarker. As mentioned above, growing evidence suggests that ENO1 is upregulated in a broad range of human tumors, making it a candidate cancer biomarker. ENO1 localization on the surface of cancer cells also provides an excellent opportunity to develop small molecules with high affinity to this protein, which enables its direct targeting in the tumor surface for diagnostic imaging and therapeutics.

The diagnostic and prognostic value of ENO1 overexpression has been confirmed in several tumors (Table 1). For example, in breast cancer, enhanced ENO1 expression correlated with greater tumor size, poor nodal status, and a shorter disease-free interval (Tu et al., 2010). Patients with lung cancer overexpressing ENO1 also showed poor clinical outcomes, with shorter overall and progression-free survival, compared to low expressing patients (Chang et al., 2006; Hsiao et al., 2013). ENO1 overexpression in hepatocellular carcinoma increased with tumor de-differentiation and correlated positively with venous invasion (Takashima et al., 2005; Hamaguchi et al., 2008). These characteristics position ENO1 as a selective biomarker able to identify aggressive tumor types with high accuracy.

Alpha-enolase has several key characteristics of an ideal cancer biomarker: (1) localization in the cell surface where it can be targeted for imaging and treatment; (2) overexpression in cancer cells with low expression in normal tissues; and (3) overexpression correlating with prognosis and clinical outcomes. Thus, ENO1 can be envisioned as an excellent biomarker to guide patient management and alter disease timeline. Ultimately, ENO1 surface imaging could potentially be used to screen for occult cancers. This information could then be translated to improve prognosis and management of patients diagnosed with cancer by monitoring disease state, detecting recurrence and progression, or assessing response to therapy.

Alpha-enolase has a potent three punch combination to advance cancer progression: (1) promotes tumor glycolysis, (2) activates cancer signaling pathways, and (3) drives tumor migration, invasion, and metastasis. These unique characteristics make ENO1 a strong candidate to deliver targeted therapies to tumors overexpressing this protein, particularly those tumors expressing surface ENO1. For instance, molecular imaging of tumors guided by ENO1-specific small molecule probes could open the door to new strategies to target this protein in tumors, leading to early interventions and improved patient outcomes. Several reports have already provided pre-clinical data supporting ENO1 therapeutic targeting. As mentioned above, ENO1 depletion attenuates glycolysis, cell proliferation, EMT, migration, and invasion, and metastasis in several cancer types (Fu et al., 2015; Capello et al., 2016; Principe et al., 2017; Zhan et al., 2017; Ji et al., 2019; Sun et al., 2019). Targeting of ENO1 in combination with chemotherapy may be beneficial in patients with drug resistant cancers given, as mentioned earlier, its emerging role in chemoresistance.

There is a major need for small molecule inhibitors of ENO1. A promising inhibitor, ENOblock, has been used to target ENO1 in various disease contexts (Jung et al., 2013; Cho et al., 2017, 2019; Haque et al., 2017; Polcyn et al., 2020) but its specificity was disputed (Satani et al., 2016). Another ENO1 inhibitor was recently reported to enhance anti-multiple myeloma (MM) immunity in combination with immunotherapy in pre-clinical models (Ray et al., 2020). In addition, a novel nanoparticle-delivered peptide targeting ENO1 in combination with doxorubicin demonstrated strong antitumor activity in pre-clinical models of PCa (Wang et al., 2018).

The study of immune responses to ENO1 has sparked the development of novel immunotherapeutic strategies. For instance, treatment of lung cancer cells with anti-ENO1 monoclonal antibodies *in vitro* suppressed cell-associated plasminogen and matrix metalloproteinase activation, collagen and gelatin degradation, and cell invasion (Hsiao et al., 2013). Interestingly, adoptive transfer of these antibodies to mice resulted in their accumulation in subcutaneous tumors and inhibition of lung and bone metastases. Principe et al. (2015) reported that *in vitro* and *in vivo* blockade of ENO1 with anti-human ENO1 monoclonal antibodies reduced PDA cell migration and invasion. Further, administration of adeno-associated virus (AAV) encoding an anti-ENO1 monoclonal antibody led to a reduction of lung metastasis in mouse PDA xenografts (Principe et al., 2015). The same group developed an ENO1 DNA vaccine that significantly inhibited, although did not eradicate, tumor growth in a mouse PDA model, suggesting that the effectiveness of this vaccine could be amplified in the context of combinatorial therapies (Cappello et al., 2013, 2018). Recently, Mandili et al. (2020) demonstrated that treatment of PDA mice with combined gemcitabine chemotherapy and ENO1 DNA vaccination induced a strong CD4+ T cell antitumor activity that impaired tumor progression, compared with mice that received vaccine or gemcitabine alone.

## Conclusion

Alpha-enolase promotes cellular functions associated with tumor aggressiveness, including increased glycolysis, activation of oncogenic signaling pathways, chemoresistance, and cell proliferation, migration, invasion, and metastasis. Therefore, ENO1 can be considered an oncoprotein critical for maintaining several “hallmarks of cancer” (Hanahan and Weinberg, 2011), particularly sustained proliferative signaling, deregulated energy metabolism, apoptosis resistance, and activation of invasion and metastasis. ENO1 overexpression in a broad range of human cancers and targetability make it an attractive cancer biomarker candidate and therapeutic target. Its localization in the tumor surface, key metabolic functions, and ability to promote tumor aggressive properties could be exploited for the development of novel comprehensive cancer care modalities that combine ENO1 surface imaging with targeted therapeutic interventions.

## Author Contributions

GLO-H designed and prepared the figures and crosschecked the references for accuracy. CAC designed the overall organization of the manuscript and approved the final version. All authors contributed to the literature review and the writing and final editing of the manuscript.

## Conflict of Interest

The authors declare that the research was conducted in the absence of any commercial or financial relationships that could be construed as a potential conflict of interest.
